# What Is in the Salad? *Escherichia coli* and Antibiotic Resistance in Lettuce Irrigated with Various Water Sources in Ghana

**DOI:** 10.3390/ijerph191912722

**Published:** 2022-10-05

**Authors:** Gerard Quarcoo, Lady A. Boamah Adomako, Arpine Abrahamyan, Samuel Armoo, Augustina A. Sylverken, Matthew Glover Addo, Sevak Alaverdyan, Nasreen S. Jessani, Anthony D. Harries, Hawa Ahmed, Regina A. Banu, Selorm Borbor, Mark O. Akrong, Nana A. Amonoo, Emmanuel M. O. Bekoe, Mike Y. Osei-Atweneboana, Rony Zachariah

**Affiliations:** 1 Council for Scientific and Industrial Research-Water Research Institute, Achimota P.O. Box AH 38, Ghana; 2 Department of Theoretical and Applied Biology, Kwame Nkrumah University of Science and Technology, Private Mail Bag, University Post Office, KNUST, Kumasi, Ghana; 3 Tuberculosis Research and Prevention Center, Yerevan 0014, Armenia; 4 Kumasi Centre for Collaborative Research in Tropical Medicine, Kwame Nkrumah University of Science and Technology, Private Mail Bag, University Post Office, KNUST, Kumasi, Ghana; 5 Centre for Evidence-Based Health Care, Stellenbosch University, Stellenbosch 7602, South Africa; 6 Department of International Health, Johns Hopkins Bloomberg School of Public Health, Baltimore, MD 21205, USA; 7 International Union against Tuberculosis and Lung Disease, 75001 Paris, France; 8 Department of Clinical Research, Faculty of Infectious and Tropical Diseases, London School of Hygiene and Tropical Medicine, London WC1E 7HT, UK; 9 United Nations Children Fund, United Nations Development Programme, World Bank, World Health Organization, Special Programme for Research and Training in Tropical Diseases (TDR) WHO, 20, Avenue Appia, CH-1211 Geneva, Switzerland

**Keywords:** antimicrobial resistance, resistant genes, *Escherichia coli*, lettuce, extended-spectrum beta-lactamase (ESBL), one health, SORT IT, operational research, Ghana, West Africa

## Abstract

Introduction: Safety of the environment in which vegetables are grown, marketed and consumed is paramount as most are eaten raw. Irrigation sources include open drains and streams, which are often contaminated with human and animal waste due to poor sanitation infrastructure. In irrigated vegetable farms using such sources in Ghana, we assessed *Escherichia coli* counts, antibiotic resistance patterns and resistant genes on irrigated lettuce. Methods: A cross-sectional study was conducted between January–May 2022, involving five major vegetable farms in Ghana. Results: *Escherichia coli* was found in all 25 composite lettuce samples analyzed. Counts expressed in CFU/g ranged from 186 to 3000, with the highest counts found in lettuce irrigated from open drains (1670) and tap water using hose pipes (3000). Among all bacterial isolates, resistance ranged between 49% and 70% for the Watch group of antibiotics, 59% for the Reserved group and 82% were multidrug-resistant. Of 125 isolates, 60 (48%) were extended-spectrum beta-lactamase-producing, of which five (8%) had the *bla_TEM_*-resistant gene. Conclusions: Lettuce was contaminated with *Escherichia coli* with high levels of antibiotic resistance. We call on the Ghana Ministry of Food and Agriculture, Food and Drugs Authority and other stakeholders to support farmers to implement measures for improving vegetable safety.

## 1. Introduction

There is increased demand for the consumption of vegetables due to dietary changes and a growing understanding of their health benefits [1]. As well, vegetable production and marketing provide significant income and employment for most smallholder farmers and traders in Ghana [2]. It is thus necessary to ensure the safety of the environment in which vegetables are grown, marketed and consumed.

In Ghana, like in many African countries, growing urbanization, climatic changes and dwindling freshwater sources have made wastewater an indispensable source for irrigation of farms [3,4]. Wastewater usage can thus contribute to increased crop production and accelerate efforts toward achieving the United Nations Sustainable Development Goal (SDG 2) which is to reach a state of zero hunger [5].

**Figure 1:**
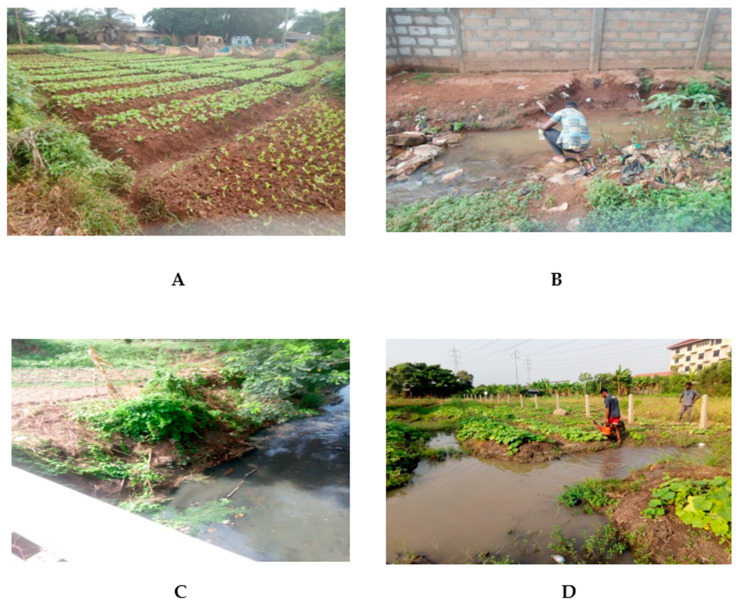
Lettuce farm (**A**), open drains used for irrigation (**B**,**C**), and water pond at a farm (**D**).

Other common irrigation sources include open drains and streams which are often contaminated with human and animal waste due to poor sanitation infrastructure [6,7,8,9]. In Ghana, open ponds and wells are also used (Figure 1).

The use of contaminated water in vegetable farming has been associated with diarrheal and helminth infections in farmers, traders and consumers [10]. Such water may also contain “antibiotic residues” which exert selective pressure, leading to the emergence and spread of antibiotic-resistant bacteria in the community [11,12,13,14]. Resistant bacteria can then spread in humans and animals via direct contact with contaminated water or through the consumption of contaminated vegetables—so-called “farm-to-fork” transmission [15,16].

The World Health Organization’s (WHO) global action plan to tackle antimicrobial resistance (AMR) emphasizes the “One Health” approach. This approach includes humans, animals, the environment, the food chain, and the interconnections between them as one entity [17]. Monitoring antibiotic resistance in food products is thus an important component of ‘One Health’. In Ghana, the focus of AMR surveillance has largely been on humans and animals with relatively little contribution from the environment such as water sources used for vegetable farming [18,19,20].

As a food safety and public health measure, the Council for Scientific and Industrial Research-Water Research Institute (CSIR-WRI) of Ghana is conducting surveillance of antibiotic resistance in bacteria found in vegetables irrigated with different water sources. Lettuce (*Lactuca sativa*) is suitable for such surveillance as it grows close to the ground, has a large surface area, is mostly eaten raw and is a widely patronized leafy green vegetable. Prevailing soil and climatic conditions are conducive for its year-round growth; it is known to be cropped up to about 9 to 10 times a year. Furthermore, farmers obtain relatively higher yearly margins from lettuce production compared to other traders, making up to about 145% returns on investment compared to other leafy vegetables [21]. Farmers will therefore be more inclined to lettuce farming. Thus, assessing the presence of *Escherichia coli* (*E. coli*) and its antibiotic resistance pattern in lettuce farms irrigated with different water sources would be most informative. This bacterium is famed for causing diarrheal outbreaks and the possible spread of antibiotic resistance, as resistant strains have been isolated from wastewater in Ghana [19,20]. It is also designated as a priority bacterium for global AMR surveillance by the WHO [22].

A PubMed search revealed a few studies on leafy vegetables from limited locations in Ghana, showing varying levels of bacterial contamination and antibiotic resistance [23,24,25,26]. However, only one study included an assessment of resistant genes [27]. What is new in this study is that we included sites from the North and South of Ghana, tested a wider panel of antibiotics for resistance, and included molecular methods for detecting resistant genes.

## 2. Materials and Methods

### 2.1. Study Design

This was a cross-sectional study using laboratory data on lettuce samples.

### 2.2. General Setting

Ghana is located in West Africa and has a population of 30.8 million in the latest census [28], with a climate characterized by rainy and dry seasons. Accra, the capital city, has a population of about 5.4 million with over 90% of its population living in urban areas [28]. Tamale, in the Northern region, is the third largest city in Ghana with a population of about 374,744 [28]. It is the fastest-growing city in Ghana, with concomitant pressure on sanitation and water resources [29].

Vegetable farming is extensive in peri-urban areas in Ghana with estimated vegetable production sites of 162 hectares (ha) and 42 ha in Greater Accra and Tamale, respectively [2,30]. The absence of a quality control system on farm produce poses a contamination risk to products sent to the market.

### 2.3. Specific Setting and Study Sites

**Figure 2:**
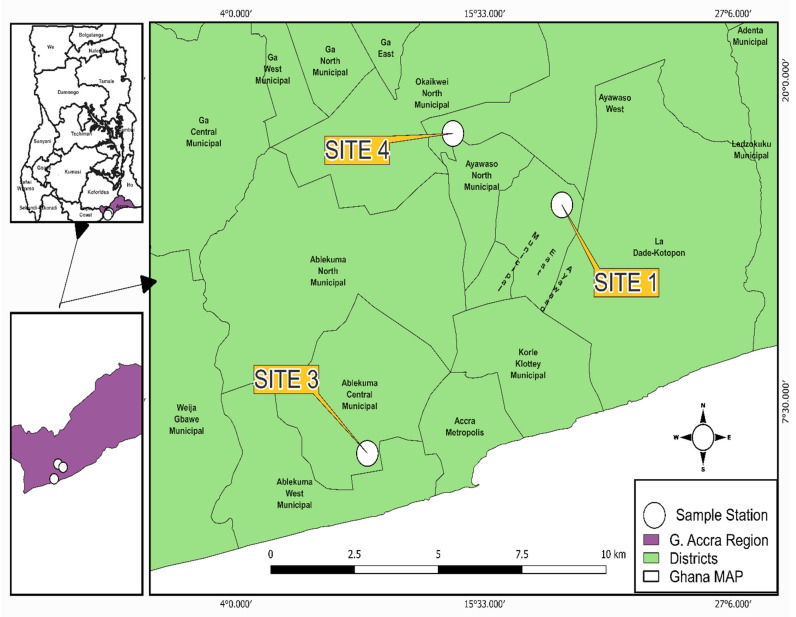
Lettuce sampling sites in Greater Accra, Ghana.

**Figure 3:**
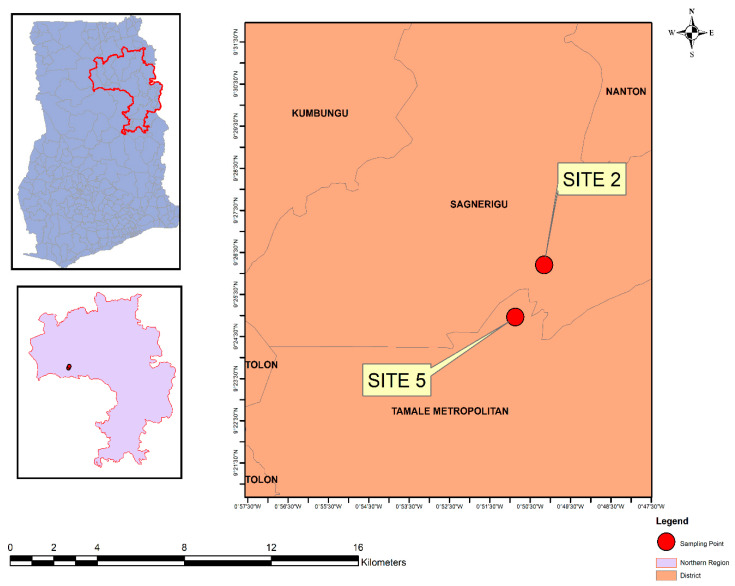
Lettuce sampling sites in Tamale, Northern Region, Ghana.

The study was conducted in Accra, Greater Accra region (Figure 2) and Tamale (Northern region) (Figure 3). The vegetable farming sites in the study included three urban sites in Accra and two in Tamale (names withheld to prevent potential social harm). These major sites often use open drains, stream water, swamps, ponds and tap water. Vegetables cultivated include lettuce, cabbage and spring onions.

The methods for irrigating lettuce are variable, depending on the available water source at the time. Most commonly, irrigation is carried out using watering cans, hoses and sprinklers.

Once matured, farm vegetables are collected by the farmers into sacks and sold to traders, who in turn sell them on the open market or directly to local restaurants.

### 2.4. Sample Collection and Bacterial Identification

Lettuce samples were collected once a month from each of the study sites. Samples were collected just before they were harvested for sale [31]. Three matured lettuce samples were randomly collected at each farm site into sterile whirl pack bags and transported to the laboratory in a cold box. At least three lettuce samples per farm site were mixed together as a composite and a total of 25 composite lettuce samples were constituted for analysis.

In the laboratory, 50 g of lettuce from each study site was weighed into a sterile bag, and 450 mL phosphate-buffered saline solution was then added and shaken vigorously. The surface of each lettuce was gently massaged through the bags before being processed and analyzed for *E. coli.*

Following a ten-fold serial dilution, the supernatant from all samples was analyzed using membrane filtration with Tryptone Bile X-glucuronide medium (TBX) (Oxoid, United Kingdom) for *E. coli*. Inoculated plates were incubated at 37 °C for 24 h [32]. *E. coli* was counted and reported as colony-forming units/gram (CFU/g). All the *E. coli* isolates were confirmed using Matrix-Assisted Laser Desorption/Ionization–Time of Flight Mass Spectrometry (MALDI-TOF MS) (Bruker MALDI Biotyper, Billerica, MA, USA).

### 2.5. Antibiotic Susceptibility Testing

Five presumptive colonies were randomly selected from each plate and sub-cultured on nutrient agar for antibiotic susceptibility testing using the Kirby Bauer Disc Diffusion method according to the Clinical Laboratory Standards Institute guidelines (CLSI) [33]. Zones of inhibition were measured in millimeters and recorded for the selected antibiotics. Antibiotics tested included those in the CLSI guidelines and those recommended for treatment of infections caused by *E. coli.* These included Ciprofloxacin 5 µg (Fluoroquinolones); Gentamicin 10 µg (Aminoglycosides); Cefuroxime 30 µg (Second-generation cephalosporins); Trimethoprim–sulfamethoxazole 1.25/23.75 µg (Sulfonamide–trimethoprim combinations); Amoxicillin/clavulanate 20/10 µg (β-lactam combination); Aztreonam 15 µg (Monobactam); Ceftriaxone 30µg (third-generation cephalosporins), Ertapenem 10µg (Carbapenem) and Chloramphenicol 30 µg (Amphenicols) (Becton Dickenson^TM^).

For phenotypic detection of Extended-Spectrum Beta-Lactamase (ESBL)-producing *E. coli* (ESBL-Ec), the five colonies from each plate were also cultured on TBX supplemented with 4 µg/mL cefotaxime (TBX/CTX) [34]. Presumptive ESBL-Ec which grew on TBX/CTX plates were confirmed using the double-disc diffusion method; Cefotaxime 30 μg/Clavulanic Acid 10 µg, Cefotaxime 30 µg, Ceftazidime 30 µg, and Ceftazidime 30 μg/Clavulanic Acid 10 µg (Becton Dickenson^TM^) and these were done in accordance with CLSI guidelines [33]. Cultured plates were incubated at 37 °C for 18–24 h [32]. Zones of inhibition were measured in millimeters and recorded. Positive ESBL-Ec isolates with ≥ 5 mm increase in the inhibition zone for Ceftazidime (30 μg) ± clavulanic acid (10 μg) and Cefotaxime (30 μg) ± clavulanic acid (10 μg) were subsequently plated on Nutrient Agar for the detection of ESBL genes.

### 2.6. Identification of Resistant Genes by Molecular Methods

DNA extraction was carried out on the presumptive ESBL isolates using Quick Zymo DNA extraction kits in accordance with the manufacturer’s instructions [35].

ESBL resistance genes (*bla*_*TEM*_, *bla*_SHV_, and *bla*_CTX-M_) were then detected using a modified polymerase chain reaction (PCR) assay from previous studies [36,37] with the Eppendorf Master Cycler (Hamberg, Germany). Each PCR reaction mix had a total volume of 12 μL, containing 5 µL of master mix (Sybr Green), 4.6 µL of nuclease-free water, 0.4 µL of optimized specific primer and 2 µL of DNA template. The primer sequence for the detection of ESBL genes in *Escherichia coli* isolates was obtained as per previous studies [36,37]. The cycling conditions were: 95 °C for 3 min, 45 cycles of 95 °C for 1 min, 56.1 °C (*bla_TEM_*)/70 °C (*bla*_SHV_, and *bla*_CTX-M_) for 1 min, 72 °C for 1 min and final extension at 72 °C for 10 min.

Aliquots of the PCR products were loaded on a 2% agarose gel and separated by electrophoresis. The DNA bands were then visualized by ethidium bromide staining using a UV illuminator (Benchtop Variable Transilluminator, Cambridge, UK) for the gel documentation and DNA fragment characterization.

### 2.7. Quality Control Procedures

Negative controls were included using sterile distilled water for all analyses. The reference organism *E. coli* ATCC 25922, was used as a positive control following CLSI guidelines. Phocine herpes virus (PhHV) was used as internal process control for DNA extraction [38]. PCR products with sizes 516, 560 and 383 base pairs were deemed positive for -*TEM, -CTX-M* and -*SHV,* respectively [36,37] when compared with a 50 base pair molecular gene marker.

### 2.8. Study Inclusion and Period

Mature lettuce samples were randomly collected and analyzed between January and May 2022.

### 2.9. Data Collection and Analysis

Data variables included: study region (North/South), study site, sample ID; sample collection date; water source at the time of sample collection; bacterial counts; antibiotic types; antibiotic sensitivity; and resistant genes.

Information on sample collection points, sample sources, bacterial loads and resistant profiles were entered into a laboratory register and then transferred to a Microsoft (MS) Excel database kept in the laboratory computer. The principal investigator and a trained data assistant entered the data. To ensure data validation, all data in the MS Excel file were cross-checked with the raw data from the laboratory register.

Bacterial counts were expressed using medians and ranges. The Kruskal–Wallis test was used to assess differences between bacterial counts per water source used for irrigation. Resistance profiles were reported using descriptive statistics. Resistance to antibiotics was categorized using the WHO Access, Watch, Reserve (AWaRe) classification [39]. All data analysis was performed using the Statistical Package for Social Science (SPSS) software (IBM version 21.0: IBM Corp, Armonk, NY, USA).

## 3. Results

### 3.1. E. coli Counts in Lettuce Irrigated from Different Water Sources

*E. coli* was found in all lettuce samples, irrespective of the water source used for irrigation. Table 1 shows counts of *E. coli,* ranging between 186 and 3000 CFU/g with the highest counts found in lettuce irrigated with water from open drains (1670 CFU/g) and lettuce sprayed with tap water using hosepipes (3000 CFU/g). Absolute counts varied between 37 and 600,000 CFU/g. There were no statistically significant differences in bacterial counts between lettuce samples irrigated from different irrigating water sources (*p*-value = 0.25).

### 3.2. Antibiotic Resistance Patterns and Resistant Genes

Table 2 shows antibiotic resistance patterns of *E coli* in lettuce in relation to the water sources used for irrigation. Resistance levels were between 49% and 70% for the Watch group of antibiotics and 59% for aztreonam (Reserve antibiotic group). The level of multidrug resistance involving at least one antibiotic from ≥3 antibiotic classes was 82%. Of the 125 isolates, 60 (48%) tested positive for ESBL, of which 5 (8%) had the *bla_TEM_*-resistant gene, and all were in lettuce irrigated with water from open drains.

## 4. Discussion

The findings of this study are important since they highlight the risk of acquiring and transmitting diarrheal infection within the community with a potential risk of outbreaks. Irrespective of the source of water used for irrigation, this study shows high *E. coli* counts in lettuce which could result from the irrigating water source and the surrounding soils [31,40]. This poses a risk for human consumption. The majority of these bacteria exhibited multidrug resistance to antibiotics, including those in the Watch and Reserve categories. This agrees with the high *E. coli* counts reported in a similar study carried out on lettuce in Accra, although antibiotic resistance patterns were not assessed at that time [31]. Similarly, multidrug-resistant *E. coli* in lettuce was reported in two other studies from Kumasi and Tamale [24,25]. Moreover, the presence of ESBL-Ec isolates on the lettuce vegetables concurs with similar studies conducted in South Africa [41], Germany [42], the Netherlands [43] and Manilla [44]. This supports the assertion that leafy green vegetables such as lettuce serve as reservoir for multidrug-resistant *E. coli* [41]. Although, the proportion of the *bla_TEM_* gene was lower, its detection in the *E. coli* isolated from lettuce is a public health concern. This gives an indication that the gene is spreading into other areas of the food value chain, as it has been detected in raw meat sold in Ghana [36].

Conversely, the high proportion of multidrug-resistant *E. coli* implies that those who contract diarrheal disease may not respond to routine antibiotic treatment, and intestinal bacterial flora may acquire antibiotic resistance through plasmid-mediated transmission. Such resistance may spread further within the population with its public health implications [8,9,45,46]. The findings thus serve as a call for increased monitoring and surveillance of antibiotic-resistant bacteria (ARBs) and antibiotic-resistant genes (ARGs) in vegetables as well as irrigation water.

There were several strengths to this study. First, it involved multiple sites from the North and the South of Ghana. In addition, microbiological analysis was conducted in the laboratory of the Water Research Institute where there is considerable expertise in laboratory control measures, data entry and validation systems. Finally, STROBE (Strengthening the Reporting of Observational Studies in Epidemiology) guidelines for ensuring the quality of reporting of observational studies in epidemiology were adhered to [47].

The main study limitation was that we could not apply comparative analysis of seasonal patterns due to our inability to capture data throughout the entire rainy and dry seasons. Furthermore, we did not test for *E. coli* in the irrigation water sources. Thus, we were unable to identify the exact source of contamination of lettuce, which might have been from water, soil and/or manure. Both of these aspects merit further research.

This study has a number of important policy and practice implications. We describe these as “Inform, Educate, Protect, and Act”. Farmer and consumer communities should be *informed* about the presence of resistant bacteria in lettuce and the potential risks associated with handling or consuming such produce without proper washing and disinfection. The community at large should also be *educated* on the importance of properly washing vegetables with clean water and further disinfection with vinegar or mild chlorinated water whenever possible to destroy bacteria [48].

In terms of *protection*, farmers should avoid direct contact with potentially contaminated water, soil and lettuce through the use of protective wear (e.g., gloves and gumboots). Where possible, the Ministry of Agriculture should provide these items in the interest of public health safety, and where this is not feasible, farmers should be empowered to invest in these simple measures for personal safety.

In terms of *Act*, there are three areas for potential action. The first is to encourage farmers to improve or adhere to good agricultural practices; for instance, they should consider the feasibility of using the drip or furrow methods for irrigation which reduces or eliminates contact of lettuce with contaminated, soil or manure [49].

The second is for regulatory bodies such as the Public Utility and Regulatory Commission (PURC) and the Food and Drugs Authority (FDA) to conduct an urgent assessment of the quality of the public water supply system since the highest bacterial counts were found in lettuce irrigated with tap water using hosepipes. While it is understandable that lettuce watered from open drains would have high loads of *E. coli*, it is surprising that high bacterial counts were found in lettuce irrigated with tap water from hosepipes. We do not know whether this is a reflection of contamination of the public water supply system, whether the water spray from the hosepipe resulted in contaminated soil being scattered on lettuce through the splash effect, or whether it is due to the direct application of manures [31,45,50].

Finally, while the standard limit of *E. coli* is <20 CFU/g for fresh vegetables in other jurisdictions such as England, Canada and New Zealand [2,51], there exists no such national microbiological reference for monitoring the safety of fresh leafy vegetables in Ghana, except for ready-to-eat foods which include salads [52]. Having such standards would help to better monitor and implement product safety measures for leafy vegetables to safeguard the health of consumers. This is an area to be considered by the Ghana Standards Authority (GSA), the Environmental Protection Agency (EPA) and the FDA.

## 5. Conclusions

This study shows that lettuce irrigated with different water sources from both the North and South of Ghana is contaminated with *E. coli.* Bacteria isolates were predominantly multidrug-resistant and the *bla_TEM_*-resistant gene was also detected. These findings highlight the need to increase AMR surveillance in this area and serves as a wake-up call to the Ministry of Food and Agriculture, the FDA and other relevant stakeholders to support farmers to produce safe vegetables in improved and hygienic environmental conditions.

## Figures and Tables

**Table 1:** Bacterial counts (CFU/g) of *Escherichia coli* in lettuce collected from vegetable farms in Ghana (January–May 2022).

Sample Sites	Irrigation Water Source	*Escherichia coli* Counts	
		Median (CFU/g)	Range
Site 1, Site 2	Open drain	1670	56–600,000
Site 3	Multiple sources (drain, pond, well)	280	37–144,000
Site 4	Tap water flowing to open ponds	186	72–1180
Site 5	Tap water using hose pipes	3000	220–14,880
**Total**		5136	385–760,060

CFU/g—Colony-Forming Unit per gram.

**Table 2:** Antibiotic resistance of *Escherichia coli* in lettuce collected from vegetable farms in Ghana, (January–May 2022).

	Resistant Isolates in Lettuce Irrigated with Different Water Sources									
	Open Drain		Multiple Open Sources (Drain, Pond, Well)		Tap Water Flowing to Open Ponds		Tap Water Using Hose Pipes		Total	
	Site 1, 2		Site 3		Site 4		Site 5			
AWaRe Categories	*n*	(%)	*n*	(%)	*n*	(%)	*n*	(%)	*n*	(%)
Total	50		25		25		25		125	
***Access antibiotics***										
Gentamicin 10 µg	13	(26)	7	(28)	1	(4)	6	(24)	27	(22)
Chloramphenicol 30 µg	31	(62)	23	(92)	12	(48)	17	(68)	83	(66)
Trimethoprim–sulfamethoxazole 1.25/23.75 µg	46	(92)	24	(96)	9	(36)	18	(72)	95	(76)
Amoxicillin/Clavulanate 20/10 µg	44	(88)	22	(88)	21	(84)	22	(88)	96	(77)
***Watch antibiotics***										
Ceftriaxone 30 µg	35	(70)	19	(76)	13	(52)	19	(76)	86	(69)
Ciprofloxacin 5 µg	20	(40)	19	(76)	6	(24)	16	(54)	61	(49)
Cefuroxime 30 µg	34	(68)	17	(68)	14	(56)	20	(80)	85	(68)
Ertapenem 10µg	27	(54)	25	(100)	17	(68)	18	(72)	87	(70)
***Reserve antibiotics***										
Aztreonam 15 µg	28	(56)	14	(56)	18	(72)	14	(56)	74	(59)
***Multidrug resistance*** (≥3 antibiotic classes)	38	(76)	25	(100)	18	(72)	21	(84)	102	(82)

## Data Availability

The data presented in this study are available on request from the corresponding author. The data are not publicly available as it is a part of an on-going study.
